# Factors Relating to Coagulation, Fibrinolysis and Hepatic Damage After Liver Resection

**DOI:** 10.1155/1993/91843

**Published:** 1993

**Authors:** Atsushi Oguro, Hiroki Taniguchi, Takeshi Daidoh, Akiyoshi Itoh, Nobuhiro Tsukuda, Toshio Takahashi

**Affiliations:** 1 The First Department of Surgery Kyoto Prefectural University of Medicine Kyoto Japan; 2 First Department of Surgery Kyoto Prefectural University of Medicine Kawaramachi-Hirokoji, Kamigyo-ku Kyoto 602 Japan

## Abstract

A survey of the blood of twenty-two patients who had undergone hepatic resection was performed. Serum levels of *α*-2 plasmin inhibitor-plasmin complex initially decreased from 1.58 ± 0.31 *μ*g/ml on the preoperative day (PREOP), to 0.92 ± 0.14 *μ*/ml on the first postoperative day (POD 1), and then increased to 3.13 ± 0.92 *μ*g/ml on the seventh postoperative day (POD 7) (mean ± SE)). Thrombin-anti-thrombin III complex (14.2 ± 4.3 ng/ml on PREOP and 26.0 ± 4.1 ng/ml on POD 7 (mean ± SE)) and D-dimer (335 ± 96 ng/ml on PREOP and 1859 ± 258 ng/ml on POD 7 (mean ± SE)) increased in the early postoperative stage. The level of 6-keto-prostaglandin F1*α* increased after the operations (from 13.2 ± 1.8 pg/ml on PREOP to 37.8 ± 12.8 pg/ml on POD 7 (mean ± SE)). The level of thromboxane B-2 decreased at first, and then gradually increased and returned to its preoperative level on POD 7 (144.7 ± 43.8 pg/ml on PREOP, 57.6 ± 27.5 pg/ml on POD1 and 152.5 ± 58.4 pg/ml on POD 7 (mean ± SE)). Superoxide dismutase activity increased at first, and then gradually decreased, postoperatively (2.8 ± 0.5 NU/ml on PREOP, 4.8 ± 0.8 NU/ml on POD1 and 2.6 ± 0.3 NU/ml on POD 7 (mean ± SE)). That is, biodefensive reactions which protect patients against the shift to disseminated intravascular coagulation (DIC) were inferred with by the increase in antiplatelet aggregation, despite the activation of coagulation and fibrinolytic mechanisms after hepatic resection.


*HPB Surgery*, 1993, Vol. 7, pp. 43–49
Reprints available directly from the publisher
Photocopying permitted by license only
© 1993 Harwood Academic Publishers GmbH
Printed in the United States of America

### FACTORS RELATING TO COAGULATION, FIBRINOLYSIS AND HEPATIC DAMAGE AFTER LIVER RESECTION

ATSUSHI OGURO, HIROKI TANIGUCHI, TAKESHI DAIDOH, AKIYOSHI ITOH, NOBUHIRO TSUKUDA and TOSHIO TAKAHASHI
*From the First Department of Surgery, Kyoto Prefectural University of Medicine, Kyoto, Japan*

*(Received 10 September 1992)*

A survey of the blood of twenty-two patients who had undergone hepatic resection was performed. Serum levels of $\alpha$-2 plasmin inhibitor-plasmin complex initially decreased from 1.58 ± 0.31 $\mu$g/ml on the preoperative day (PREOP), to 0.92 ± 0.14 $\mu$/ml on the first postoperative day (POD 1), and then increased to 3.13 ± 0.92 $\mu$g/ml on the seventh postoperative day (POD 7) (mean ± SE)). Thrombin-anti-thrombin III complex (14.2 ± 4.3 ng/ml on PREOP and 26.0 ± 4.1 ng/ml on POD 7 (mean ± SE)) and D-dimer (335 ± 96 ng/ml on PREOP and 1859 ± 258 ng/ml on POD 7 (mean ± SE)) increased in the early postoperative stage. The level of 6-keto-prostaglandin F1$\alpha$ increased after the operations (from 13.2 ± 1.8 pg/ml on PREOP to 37.8 ± 12.8 pg/ml on POD 7 (mean ± SE)). The level of thromboxane B-2 decreased at first, and then gradually increased and returned to its preoperative level on POD 7 (144.7 ± 43.8 pg/ml on PREOP, 57.6 ± 27.5 pg/ml on POD 1 and 152.5 ± 58.4 pg/ml on POD 7 (mean ± SE)). Superoxide dismutase activity increased at first, and then gradually decreased, postoperatively (2.8 ± 0.5 NU/ml on PREOP, 4.8 ± 0.8 NU/ml on POD 1 and 2.6 ± 0.3 NU/ml on POD 7 (mean ± SE)). That is, biodefensive reactions which protect patients against the shift to disseminated intravascular coagulation (DIC) were inferred with by the increase in anti-platelet aggregation, despite the activation of coagulation and fibrinolytic mechanisms after hepatic resection.

KEY WORDS: Coagulation, fibrinolysis, disseminated intravascular coagulation, liver resection, platelet aggregation

### INTRODUCTION

Disseminated intravascular coagulation (DIC) is a life-threatening, hemorrhagic state where the activation of coagulation and the subsequent activation of fibrinolysis occur.

It is safe to say that patients on whom hepatectomies have been performed are in the predisseminated intravascular coagulation state because of hypercoagulation and hyperfibrinolysis after the hepatectomy. Coagulation, fibrinolysis, platelet aggregation, liver regenerative and liver destructive factors are related to the occurrence of DIC.

---

Address correspondence to: Atsushi Oguro, M.D., First Department of Surgery, Kyoto Prefectural University of Medicine, Kawaramachi-Hirokoji, Kamigyo-ku, Kyoto, 602, Japan

This paper describes serological changes after a hepatectomy through the measurement of serial levels of several substances that behave as coagulating or fibrinolytic factors, platelet aggregation regulators or that correlate with liver regeneration and destruction in plasma before and after hepatic resection.

### PATIENTS AND METHODS

Twenty-two patients underwent liver resections at Kyoto Prefectural University of Medicine Hospital between July 1989 and January 1990. There were 16 men and 6 women. Their ages ranged from 40 to 77 years with a median of 62 years. They were classified by diseases: 12 patients had hepatocellular carcinomas, 6 had metastatic liver cancer, and 4 had malignant biliary tumors. Ten patients had liver cirrhosis and 12 did not. Hepatectomies or further resections were performed on 7 patients, 3 of whom had cirrhotic livers. Segmentectomies were done on 3 patients, one of whom was cirrhotic. Six patients, all having liver cirrhosis, underwent subsegmentectomies. Six patients underwent partial resections, two of whom were cirrhotic. The median of operating time was 285 minutes, with a range of 130 to 516 minutes. The median of blood loss was 1241 grams, with a distribution from 300 to 6021 grams. The median requirement for blood transfusion was 0 units and the average was 3.1 units, with a maximum of 11.5 units. Seven patients received 0.5 to 1.5 units of fresh whole blood. The rest of the transfused blood was concentrated red cells (CRC). The median of fresh frozen plasma (FFP) used was 5 units, with a maximum of 22 units.

Blood samples were taken from these patients before their operations, on postoperative day (POD) 1, POD 3 and POD 7, and then divided to measure prothrombin time (PT), plasma fibrinogen, serum fibrinogen/fibrin degradation products (FDP) (normal value $< 5$ $\mu$g/ml), serum thrombin-anti-thrombin III complex (TAT), serum $\alpha$2-plasmin inhibitor-plasmin complex (PIC), serum D-dimer (DD), serum 6-keto-prostaglandin F1$\alpha$ (6-keto-PG1$\alpha$), serum thromboxane B-2 ($TXB_2$), serum superoxide dismutase (SOD) activity and serum prostaglandin $E_2$ ($PGE_2$). Serum total bilirubin, serum glutamic oxalacetic transaminase (S-GOT), serum glutamic pyruvic transaminase (S-GPT), platelet count, and the preoperative value of the 15 minute indocyanine green retention test (ICG R15), a parameter related to liver function, was also measured.

PT (normal values: 80–110%) was measured by Quick's test and indicated as prothrombin index. Plasma fibrinogen (normal values: 200–400 mg/dl) was measured using thrombin time. FDP was measured by latex fixation test. TAT (normal value $< 3.0$ ng/ml) was measured by enzyme-linked immunosorbent assay (ELISA) using Enzygmost-TAT (Hoechst Japan Co. Japan). PIC (normal value $<$ 0.8 $\mu$g/ml) was tested by the enzyme immunoassay (EIA) method using $\alpha$2-PI complex "Teijin" EIA-B (Teijin Co. Japan). DD (normal value $< 150$ ng/ ml) was assayed by ELISA using DIMERTEST EIA (Fuji Repio Co. Japan). 6-keto-PGF1$\alpha$ (normal values: 12–33 pg/ml), $TXB_2$ (normal values: 14–50 pg/ml) and $PGE_2$ (normal values $< 8.4$ pg/ml in males and $< 4.4$ pg/ ml in females) were measured by radioimmunoassay (RIA). SOD activity (normal value $< 21$ NU/ml) was determined by the nitrate-kit method (Fujisawa Pharmaceutical Co. Japan). Results were statistically evaluated by Student's *t*-test.

### RESULTS

The mean preoperative value of ICG R15 was 19.0%. The mean serum total bilirubin level on the preoperative day (PREOP), POD 1, POD 3, and POD 7 was 0.9, 2.0, 1.7, and 1.0 mg/dl, respectively. The mean serum GOT level on PREOP, POD 1, POD 3, and POD 7 was 40.4, 146.9, 160.4, 43.4 Karmen Unit (KU), respectively. The mean serum GPT level on PREOP, POD 1, POD 3, and POD 7 was 26.3, 82.0, 96.9, 36.5 KU, respectively. Mean PT value on PREOP, POD 1, POD 3, and POD 7 was 97.6, 91.9, 104.6, 96.7%, respectively. The mean plasma fibrinogen level on PREOP, POD 1, POD 3, and POD 7 was 310.6, 308.5, 534.4, 403.4 mg/dl, respectively, and the value for POD 3 was significantly higher than the value for POD 1 ($p < 0.005$). The mean serum FDP level on PREOP, POD 1, POD 3, and POD 7 was 4.49, 9.36, 17.94, 22.9 μg/ml, respectively, and no statistically significant difference was noted between the values on POD 1 and POD 3. The mean platelet count on PREOP, POD 1, POD 3, and POD 7 was 229000, 180000, 198000, 247000, respectively. The serum TAT level on PREOP, POD 1, POD 3, and POD 7 was 14.2 ± 4.3, 27.4 ± 4.0, 29.8 ± 4.1, 26.0 ± 4.1 ng/ml (mean ± standard error (SE)), respectively (Figure 1). The serum PIC level on PREOP, POD 1, POD 3, and POD 7 was 1.58 ± 0.31, 0.92 ± 0.14, 4.45 ± 1.86, 3.13 ± 0.92 μg/ml (mean ± SE), respectively (Figure 1) and the serum DD level

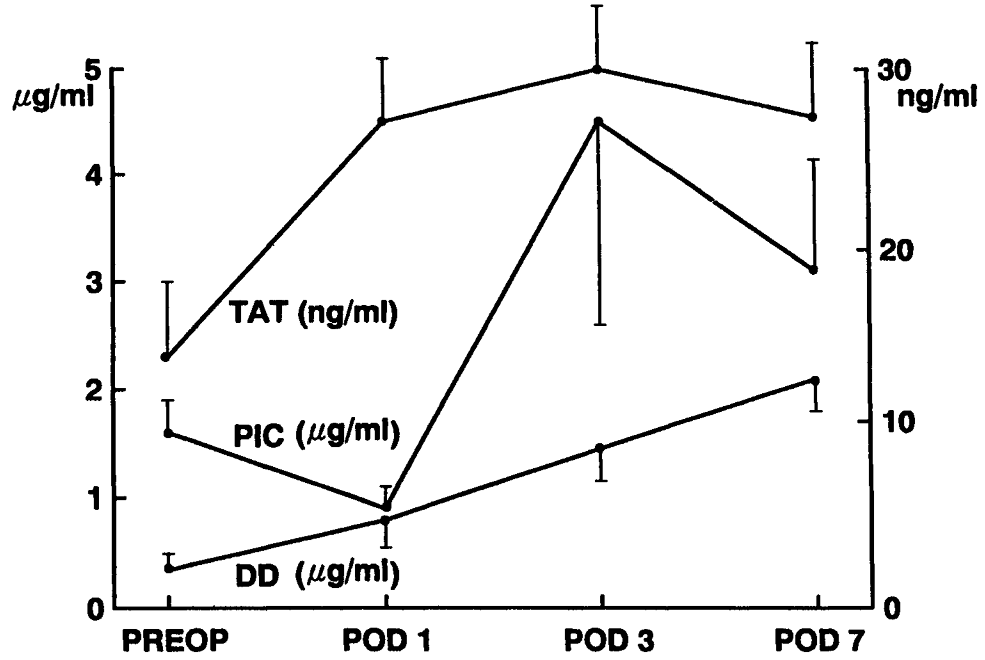

**Figure 1** The levels of serum thrombin-antithrombin III complex (TAT), α-2 plasmin inhibitor-plasmin complex (PIC) and D-dimer (DD) increased in the first operative week. Each value is the mean ± standard error (SE).

on PREOP, POD 1, POD 3, and POD 7 was 335 ± 96.5, 820 ± 175.3, 1472 ± 290.0, 1859 ± 257.7 ng/ml (mean ± SE), respectively (Figure 1). Both PIC and DD levels tended to increase postoperatively. The serum 6-keto-PGF1$\alpha$ level on PREOP, POD 1, POD 3, and POD 7 was 13.2 ± 1.8, 21.2 ± 3.7, 23.4 ± 4.2, 37.8 ± 12.8 pg/ml (mean ± SE), respectively, and the value tended to increase postoperatively. The serum $TXB_2$ level on PREOP, POD 1, POD 3, and POD 7 was 144.7 ± 43.8, 57.6 ± 27.5, 79.1 ± 27.2, 152.5 ± 58.4 pg/ml (mean ± SE), respectively (Figure 2). It can be seen that the value decreased on POD 1, and then recovered. The serum SOD activity level on PREOP, POD 1, POD 3, and POD 7 was 2.8 ± 0.5, 4.8 ± 0.8, 3.8 ± 0.7, 2.6 ± 0.3 NU/ml (mean ± SE), respectively. SOD activity peaked on POD 1 and subsequently decreased gradually. The serum $PGE_2$ level on PREOP, POD 1, POD 3, and POD 7 was 4.5 ± 0.7, 3.4 ± 0.4, 5.7 ± 1.3, 16.1 ± 8.4 pg/ml (mean ± SE), respectively, and the value exhibited a tendency to increase postoperatively.

There was, in this small group of 22 patients, no significant difference, either between patients with or without liver cirrhosis, or among patients who had undergone different types of liver resections, with regard to serum TAT, PIC, DD, 6-keto-PGF1$\alpha$, $TXB_2$ levels or SOD activity.

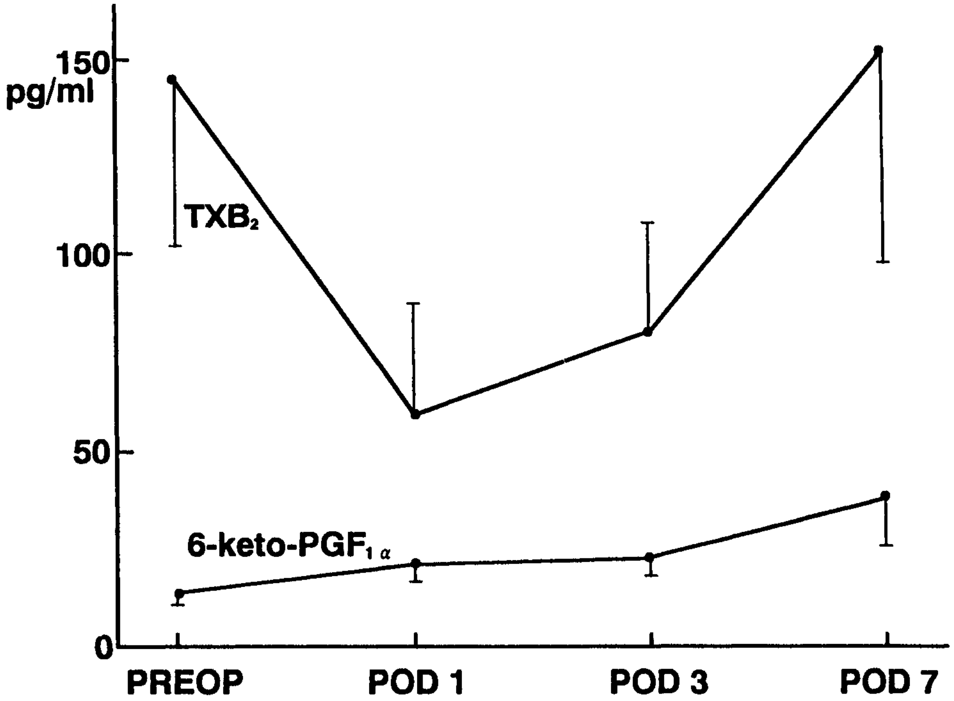

**Figure 2** The level of serum 6-keto-prostaglandin F1$\alpha$ (6-keto-PGF1$\alpha$) gradually increased after hepatic resection. The level of serum Thromboxane B-2 ($TXB_2$) decreased on postoperative day 1, and then returned to its preoperative range within one week. Each value is the mean ± standard error (SE).

### DISCUSSION

DIC is a syndrome which is characterised by extended intravascular coagulation with consumption of coagulation factors and pathological overactivation of the fibrinolysis, leading to an increased tendency to bleed. Diseases often implicated as triggers of DIC include malignant tumors, leukemia and sepsis.

In surgery, the activation of blood coagulation is inevitable and reticuloendothelial system hypofunction, or shock state, easily occurs. Therefore, it can be said that postoperative patients are in the pre-DIC state. Especially for a hepatic resection, postoperative DIC cannot be ignored because it is assumed that the remnant liver is unable to remove active coagulation factors released by extensive liver resection and massive blood transfusion[1]. Moreover, Almersjoe *et al.* reported that the coagulation defect occurs after extensive liver resection[2]. In that regard Tsuzuki and his associates reported that the incidence of DIC correlated with the extent of hepatic resection or blood loss. Patients in whom of two or more segments of the liver were resected, or more than 4000g of blood was lost had a statistically higher incidence of DIC[3]. Bengmark *et al.* found that in rats, defects in hemostasis were produced by antibiotics after liver resection[4]. In the present study, 7 patients underwent hepatectomies or further resections and 2 patients lost more than 4000g of blood. However, neither these 9 patients nor any of the other 13 patients needed a transfusion of platelets, or the administration of anti-thrombin III or heparin.

During the course of blood coagulation thrombin is produced in blood, and is presumed to quickly form a complex with anti-thrombin III. Therefore the measurement of TAT has the same significance as obtaining the blood value of thrombin, which cannot be detected directly. The serum TAT level increase in cases of disease accompanied by hypercoagulation, DIC, malignant tumors, pulmonary embolism and phlebothrombosis. Thus, TAT plays a role in the detection of hypercoagulation and DIC[5–7]. Plasmin, which is regarded as the one of the most important fibrinolytic factors, has so short a half-life that it cannot be measured. However, $\alpha$2-plasmin inhibitor, which is the most important plasmin inhibitor, instantly inhibits plasmin which has just been produced. Thus, the formation of PIC reflects the production of plasmin. The detection of an increase of PIC in the blood is useful in the diagnosis of DIC in its early stages. Quantification of PIC and a study of the behavior of the $\alpha$2-plasmin inhibitor have been performed[8,9]. Fibrinogen/fibrin degradation product (FDP) consists of several fragments, and in normal fibrinogenolysis, the D fragment forms monomers. On the other hand, cross-linked fibrin produces DD during proteolysis. Therefore DD can be found only during secondary fibrinolysis, and thus its measurement is useful for the diagnosis of DIC, including DIC in its early phase. DD and its surroundings have been studied previously[10,11]. In this study, although PIC had an initial decrease, the level of not only TAT, but also DD, increased in the postoperative seven days. Some patients may be in the pre-DIC state after hepatic resection because coagulation and fibrinolysis progress simultaneously.

Thromboxane A-2 ($TXA_2$) promotes platelet aggregation, and its stable metabolite, $TXB_2$, provides a good indication of the $TXA_2$ concentration[12,13]. Prostaglandin $I_2$ ($PGI_2$) inhibits platelet aggregation by acting antagonistically to $TXA_2$, and the stable metabolite of $PGI_2$, 6-keto-PGF1$\alpha$, is usually measured to assess the rate of $PGI_2$ synthesis[12]. In this study, the level of 6-keto-PGF1$\alpha$

increased after the hepatectomies whereas the $TXB_2$ level decreased initially, then increased gradually and finally returned to its preoperative value on POD 7. However, the results do not establish the presence of anti-aggregatory activity because $PGI_2$ production did not proceed in the absence of platelet adhesion or aggregation *in vivo*.

SOD eliminates superoxides, which are regarded as DIC-aggravating substances[14]. Moreover SOD may be considered platelet inhibitor because Parnham *et al.* reported that platelet activating factor induced the oxidative burst in guinea-pig macrophages[15]. In this study the SOD activity increased at first, and then decreased gradually after the operation but against our expectation, its value always remained within normal range. Involvement of prostaglandins in liver regeneration after a partial hepatectomy has been suggested[16] and the possibility that $PGE_2$ and prostaglandin F2$\alpha$ (PGF2$\alpha$) are liver regeneration factors has been reported[17]. Furthermore, a possible role of $PGE_2$ as platelet inhibitor[18,19] and fibrinolytic activator[20] has been suggested. Though the serum SOD activity remained within its normal value, the possibility was suggested that, in the pre-DIC state, the production of SOD increased in order to eliminate free radicals and that the aggravation of DIC progressed until the elimination of free radicals was completed. Similarly, the production of $PGE_2$ increased in order to promote liver regeneration after hepatic damage.

It is thought that because of the decrease of coagulating function due to hyposynthesis in patients with liver cirrhosis, hypocoagulation after hepatic resection may occur and DIC is more likely to occur than in patients without liver cirrhosis. However, in this study no significant differences between the cirrhotic group and the non-cirrhotic one were found in serum TAT, PIC, DD, 6-keto-PG1$\alpha$, $TXB_2$, $PGE_2$ or in SOD activity. This may be due to the efficacy of postoperative intensive care, the administration of FFP, liver protective therapy, restriction of water intake, etc. which could have prevented DIC from occurring. In order to prevent pneumonia during postoperative intensive care, patients were encouraged to refrain from lying down for long periods and epidural anesthesia was used for the peri- and postoperative periods. In order to prevent bleeding from the GI-tract, $H_2$-receptor antagonist, sulpiride, aluminium hydroxide gel and magnesium hydroxide were administered postoperatively. Furthermore, it is important to decide on the extent of hepatic resection in proportion to liver function, and to reduce blood loss as much as possible.

In conclusion, the activation of coagulation and fibrinolytic mechanisms may be induced after hepatic resection. However, this state may be improved by an increase in anti-platelet aggregation. We inferred the existence of biodefensive reactions which protect patients against the shift to DIC.

### *References*

1. Deykin, D. (1966) The role of the liver in serum-induced hypercoagulability. *J. Clin. Invest.*, **108**, 605–608
2. Almersjoe, O., Bengmark, S. and Engevik, L. (1967) The coagulation defect after extensive liver resection in man. *Scand. J. Gastroent.*, **2**, 204–208
3. Tsuzuki, T., Toyama, K. and Nakayasu, K. (1990) Disseminated intravascular coagulation after hepatic resection. *Surg.*, **107**, 172–176
4. Bengmark, S., Goeransson, G. and Zoukas, E. (1981) Defects in hemostasis produced by antibiotics. *Eur. Surg. Res.*, **13**, 290–298

5. Pelzer, H., Egbring, R. and Seitz, R. (1987) Thrombin-antithrombin III Complex-A new parameter for detection of thrombotic states. *Thromb. Heamost.*, **58**, 237
6. Hoek, J., Lamping, R. and Sturk, A. (1987) Thrombin-antithrombin (TAT) III complexes-A study in consecutive patients suspected of DIC. *Thromb. Haemost.*, **58**, 237
7. Seitz, R., Praetorius, G. and Blanke, H. (1987) Increase of thrombin-antithrombin III (TAT) complex plasma levels in thromboembolic diseases during thrombolysis. *Thromb. Haemost.*, **58**, 237
8. Harpel, P.C. (1981) $\alpha$2-Plasmin inhibitor and $\alpha$2-macroglobulin-plasmin complexes in plasma. *J. Clin. Invest.*, **68**, 46–55
9. Aoki, N., Moroi, M. and Matsuda, M. (1977) The behavior of $\alpha$2-plasmin inhibitor in fibrinolytic states. *J. Clin. Invest.*, **60**, 361–369
10. Amiral, J., Plassart, V. and Minard, F. (1986) Measurement and clinical relevance of D. dimer by ELISA. In: *Fibrinogen and its derivatives*, edited by G. Mueller-Bergenhaus *et al.* pp. 285–290. Amsterdam: Elsevier Science Publishers (Biomedical Division)
11. Olexa, S.A. and Budzynski, A.Z. (1979) Primary soluble plasmic degradation product of human cross-linked fibrin. Isolation and stoichiometry of the (DD)E complex. *Biochem.*, **18**, 991–995
12. Maurin, N. (1986) Routine measurement of thromboxane $B_2$ and the prostacyclin metabolite 6-keto-prostaglandin F1$\alpha$ in plasma. *Arzneim-Forsch Drug Res.*, **36**(II), 1174–1179
13. Hornch, A., Kriff, C. and Briety, J. (1982) Radioimmunoassay measurement of thromboxane $B_2$ in human plasma and urine. In: *Prost. Leukotr. Med.*, edited by D.F. Horrobin and M. Karmazyn, vol. 8, pp. 467–480. Edinburgh: Churchill Livingstone Med. J.
14. Yoshikawa, T., Seto, O. and Itani, K. (1985) Endotoxin-induced disseminated intravascular coagulation and shock, and their relationship with oxygen-derived free radicals. In: *Oxygen Free radicals in shock.*, edited by Novelli, Ursini, pp. 125–136. Florence: Int. Workshop
15. Hartung, H.P., Parnham, M.J. and Winkelmann, J. (1983) Platelet activating factor (PAF) induces the oxidative burst in macrophages. *Int. J. Immunopharmac.*, **5**, 115–121
16. Miura, Y. and Fukui, N. (1979) Prostaglandins as possible triggers for liver regeneration after partial hepatectomy. A review. *Cellular & Molecular Biology*, **25**, 179–184
17. MacManus, J.P. and Braceland, B.M. (1976) A connection between the production of prostaglandins during liver regeneration and the DNA synthetic response. *Prostaglandins*, **11**, 609–620
18. Parnham, M.J. and Bittner, C. (1983) Chemiluminescence from mouse resident macrophages: characterization and modulation by arachidonate metabolites. *Immunopharmac.*, **5**, 277–291
19. Parnham, M.J. and Bittner, C. (1984) Differential effects of prostaglandins and indometacin on the macrophage oxidative burst. *Agents and Actions*, **15**, 46–47
20. Crutchley, D.J., Conanan, L.B. and Maynard, J.R. (1982) Stimulation of fibrinolytic activity in human skin fibroblasts by prostaglandins $E_1$, $E_2$ and $I_2$. *J. Pharmacol. Exp. Ther.*, **222**, 544–549

*(Accepted by S. Bengmark 11 September 1992)*

